# Dual-tracer autoradiographic analysis of glucose metabolism and hypoxia in orthotopic and PDX tumor models

**DOI:** 10.2340/1651-226X.2025.44002

**Published:** 2025-08-30

**Authors:** Morten Busk, Martin K. Thomsen, Jens Overgaard, Martin F. Berthelsen, Henrik Hager, Johan Bussink, Kim V. Hansen, Steen Jakobsen, Jacob Kinggard Lilja-Fischer, Ebbe Boedtkjer, Mikkel H. Vendelbo

**Affiliations:** a Department of Experimental Clinical Oncology, Aarhus University Hospital, Aarhus, Denmark; b Danish Centre for Particle Therapy, Aarhus University Hospital, Aarhus, Denmark; c Department of Biomedicine, Aarhus University, Aarhus, Denmark; d Department of Clinical Medicine, Aarhus University, Aarhus, Denmark; e Department of Pathology, Aarhus University Hospital, Aarhus, Denmark; f Department of Radiation Oncology, Radboud University, Nijmegen Medical Centre, Nijmegen, The Netherlands; g Department of Nuclear Medicine and PET Centre, Aarhus University Hospital, Aarhus, Denmark

**Keywords:** hypoxia, radioresistance, image-guided radiotherapy, PET, FDG, FAZA

## Abstract

**Background and purpose:**

Quantification/mapping of tumor hypoxia may guide pretreatment decision-making in radiation oncology. Hypoxia-selective positron emission tomography (PET) tracers, like ^18^F-fluoroazomycin arabinoside (FAZA), allow assessment of hypoxia, but since hypoxia stimulates glycolysis, fluorodeoxyglucose (FDG) and hypoxia-PET may provide overlapping/similar information. Clinical dual-tracer PET studies are highly complex and remain inconclusive. Accordingly, we developed dual-tracer autoradiography techniques to allow high-resolution assessment of the spatial coupling of FAZA and ^14^C-2DG (FDG-analogue), without the time-separation and co-registration-related inaccuracies intrinsic to PET.

**Patient/material and methods:**

Orthotopic lung adenocarcinomas were induced in CRISPR/*Cas9* knock-in mice. Mammary adenocarcinomas developed spontaneously in transgenic mice overexpressing ErbB2 (Her2). Patient-derived-xenografts (PDX) were established in immunocompromised mice using biopsies from oropharyngeal cancer patients. Tumor growth was followed by MRI/Caliper measurements. Mice were administered with FAZA (~40 MBq)/^14^C-2DG (37 kBq)/pimonidazole and sacrificed. Tumor cryosections were analyzed for FAZA/^14^C-2DG using dual-tracer autoradiography followed by histological stainings. Complementary autoradiograms were co-registered and covered by a square-grid (0.5 × 0.5 mm), and Pearson correlation coefficients (*R*) were calculated.

**Results/Interpretation:**

Hypoxic sub-volumes (FAZA/pimonidazole) were commonly present. A reasonable spatial overlap between FAZA and ^14^C-2DG was observed in most lung and oropharyngeal tumors with *R* typically exceeding 0.55. In the breast tumor model, the extent of overlap between FAZA and ^14^C-2DG varied widely with *R* ranging from 0.03 to 0.82, which may relate to intertumor mutational differences in this Her2+ oncogene-driven model. Our results suggest a putative role for FDG-PET to identify hypoxic foci and guide dose-escalation.

## Introduction

Hypoxic cells are present in most tumors, including squamous cell carcinomas and lung, mammary, and prostate adenocarcinomas [1, 2]. Importantly, posttreatment relapse may relate to surviving treatment-resistant hypoxic cells, as shown in the DAHANCA trials, where the radiosensitizer nimorazole, which on its own shows insignificant cytotoxicity, improved loco-regional control in patients with hypoxic HPV-negative head and neck squamous cell carcinomas (HNSCC) treated with radiotherapy [3]. In addition, hypoxia promotes tumor progression and metastasis [4]. Besides radiosensitizers, which have never gained widespread use, radiation dose-escalation to the whole tumor or hypoxic sub-volumes (dose painting) may be justified in treatment-resistant tumors [5]. Current technology allows non-uniform target dose prescription but requires reliable information on intratumoral tumor oxygenation. Hypoxia-PET using 2-nitroimidazoles like ^18^F-fluoromisonidazole (FMISO) or FAZA for treatment planning and guidance is feasible yet underutilized, possibly due to inherent weaknesses with poor inter-tissue and intratumoral image contrast. Except for head and neck squamous cell carcinomas, there is little clinical evidence that selective targeting of hypoxia improves outcome, but this could simply reflect a historical focus on that patient group [2]. Interestingly, in lung cancer patients, tumor relapse was consistently observed in patients with a tumor-to-muscle FMISO ratio above 2.0 [6], and FMISO-PET was a reliable predictor of progression-free survival irrespective of tumor size [7]. Furthermore, the development of nimorazole was largely based on the murine mammary adenocarcinoma C3H. Finally, numerous rodent studies suggest that selective targeting of hypoxic cells using hypoxia-activated cytotoxins may be beneficial in a broader selection of cancers.

Since hypoxia stimulates the use of glucose (anaerobic glycolysis) and FDG-PET/CT is a routine diagnostic scan, the identification of tumors where FDG maps hypoxia with reasonable accuracy would be of significant clinical value. Indeed, several studies have compared the spatial relationship between FDG- and hypoxia-PET maps, but results have been conflicting [8–13]. This may reflect technical issues (e.g. inaccurate image co-registration), the use of different protocols (e.g. variable time interval between the two scans), or true biological differences among included patients. Preclinical research may help us to reach more definitive conclusions on the spatial relationship between regional FDG retention and oxygenation, and whether/when FDG-PET is a suitable alternative to hypoxia-PET.

Traditional tumor models established from cell lines in immunosuppressed mice may not faithfully recapitulate human disease, since genetic drift, loss of clone variability, and phenotypic changes in energy metabolism are foreseeable after long-term culturing under nutritionally enriched conditions. To improve our understanding of the spatial relationship between FDG and hypoxia-selective tracers, we developed a dual-tracer autoradiographic methodology and tested it in clinical relevant orthotopic tumors and patient-derived xenografts (PDXs) [14–16].

## Patients/material and methods

### Tumor models

Patient-derived xenografts (PDX: AUHSCC-13 and AUHSCC-25) were established subcutaneously in severely immunocompromised CIEA/NOG GMO female mice (NOD.Cg-*Prkdc^scid^ Il2rg^tm1Sug^*/JicTac; Taconic, Denmark) from biopsies taken from patients with HPV-negative oropharyngeal squamous cell carcinoma. Subsequently, tumors were passaged in additional recipient-mice as required for experiments. For further details, see reference [16]. Orthotopic lung adenocarcinomas were induced in Cas9 transgenic mice (B6J.129(B6N)-Gt(ROSA)26Sortm1(CAG-cas9*,-EGFP)Fezh/J; Jackson Laboratories) by intranasal delivery of adeno-associated virus particles resulting in loss-of-function mutations in LKB1, KRAS, and p53. The method is described previously [15]. Mammary adenocarcinomas developed spontaneously in transgenic female mice (FVB/N-Tg(MMTVneu)202Mul/J; Jackson Laboratories) that overexpress ErbB2 specifically in breast epithelium [17, 18].

Subcutaneous PDXs and mammary adenocarcinomas were monitored using caliper measurements and used for tracer experiments when the tumor size reached ~1 cm^3^. Cas9 mice were scanned regularly, using a small-animal dedicated MRI scanner from Mediso Medical Imaging Systems, Hungary. Tumors were identified using the Nucline v2.01 software from Mediso Medical Imaging Systems.

### Radiotracer experiments

FAZA was synthetized locally at the Department of Nuclear Medicine and PET Centre as described previously [19], whereas ^14^C-2DG was obtained from American Radiolabeled Chemicals (Saint Louis, MO, USA). Pimonidazole was a gift from J.A. Raleigh (University of North Carolina, USA). When ready for experiments, mice were administered ~40 MBq FAZA (in saline) mixed with pimonidazole (0.02 mL/g equaling 60 mg/kg) as a single IP bolus (0.6–0.8 mL). Two hours later, mice were IP administered 37 kBq ^14^C-2DG in 0.2 ml saline and sacrificed by neck dislocation after an additional hour. Tumors (PDX/mammary adenocarcinomas) or lungs with tumors were dissected and frozen in pre-cooled (−40°C) iso-pentane and embedded in Tissue Tek O.C.T. for cryosectioning. In addition, brain and muscle tissues (hind leg) were collected and used as internal reference tissues for signal normalization of ^14^C-2DG and FAZA, respectively. Brains were briefly frozen superficially, halved using a scalpel along the mid-sagittal plane and refrozen. PDX and mammary tumors were cut from different layers (separated by 0.5–1 mm). Several 10 μm neighbor sections were collected from each cutting depth and placed on separate glasses, to ensure a broad selection of tissue sections without cutting artifacts (holes/folds) for autoradiographic and post-autoradiographic analyses. Lungs were cut, and whenever tumors appeared, cryosections were collected including surrounding normal lung tissue. Tissue reference sections were prepared randomly (muscle) or along the mid-sagittal axis (brain). Cryosections were exposed to BAS-SR 2025 phosphor imaging plates (Fuji Photo Film Co. Ltd, Japan) for ~2h, starting immediately after sectioning. Due to an apparatus defect, different plate reader systems, but with similar specifications, were used, which include a Fuji BAS 5000 scanner (FUJI, Japan), a FLA3000 reader (FUJI, Japan), and an Amersham Typhoon Biomolecular Imager (GE Healthcare, USA). The radioactivity distribution was obtained at a resolution of 25 μm (or 50 μm, FLA3000), which is far below the true plate resolution (see later). Tissue sections were then left for at least 36 h to allow for complete ^18^F decay and re-exposed to imaging plates for 5–6 days, to compensate for the low ^14^C-2DG dose. Due to the large difference in administered doses (~1,000-fold) of the two tracers, the first short round of autoradiography largely reflects the signal from FAZA. Based on the second round of autoradiography and correcting for differences in exposure times, it was estimated that ^14^C-2DG contributed < 2% to the FAZA signal. To preserve tissue sections prior to immunostainings and lower background contamination, the second round of autoradiography was done at -20 °C (freezer) and shielded by lead plates. Subsequently, selected tissue sections were either stained for pimonidazole as described previously [20] or H&E stained and then digitalized at a 20x magnification using a Hamamatsu NanoZoomer 2.0 HT slide scanner (Hamamatsu Photonics, Japan).

### Autoradiographic analyses

**Figure 1:**
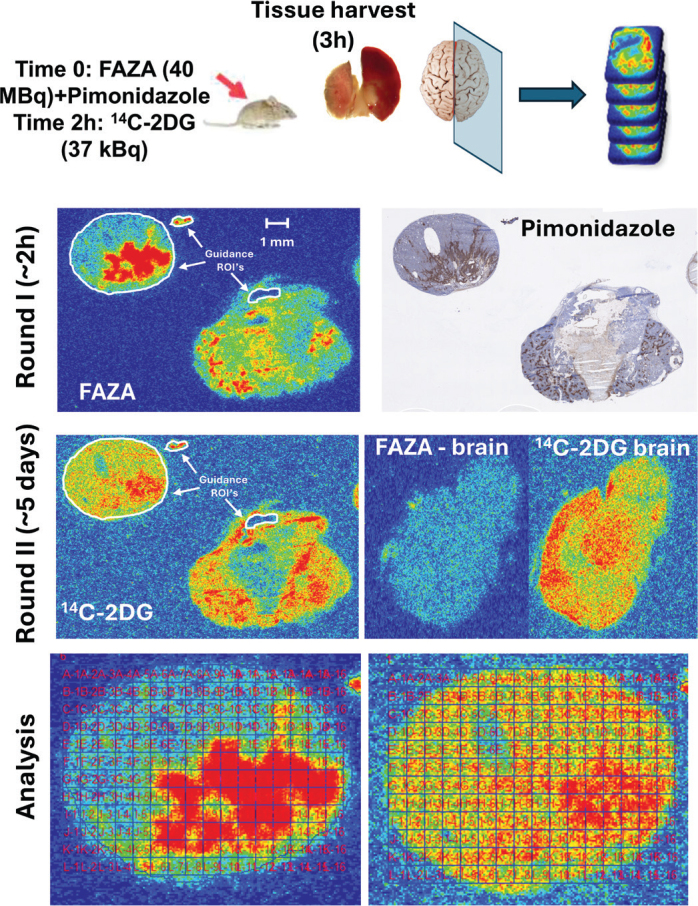
Overall study design. When tumors were ready for experiments (MRI/Caliper assessment), mice were administered a bolus of FAZA and pimonidazole. Two hours later, ^14^C-2DG was administered. After another hour, tumors and reference tissues were collected from the thigh (muscle) or the brain. Multiple neighbor cryosections were prepared from several tumor depths. In addition, tissue sections from muscle (reference for FAZA) and the mid-sagittal brain region (reference for ^14^C-2DG) were collected. Sections were immediately exposed to phosphor imaging plates (PIPs) for ~2 h to obtain the signal from the ^18^F-labeled tracer. Following complete decay of 18F, a second round of extended PIP exposure (to compensate for the low tracer dose of ^14^C-2DG) was conducted under lead shielding in a freezer, which reduces background contamination and preserves tissue quality for complementary analysis. Ultimately, selected tissue sections were H&E stained to allow accurate tumor visualization and delineation. Other sections were analyzed for pimonidazole. Finally, the spatial overlap of FAZA and ^14^C-2DG was compared by positioning a grid with a cell size of 0.5 x 0.5 mm, covering the complementary autoradiograms. To ensure highly accurate positioning of the grid on complementary autoradiograms, several so-called guidance ROIs were drawn (white color) on the first (FAZA) autoradiogram, imported into the second (^14^C-2DG) autoradiogram, and positioned similarly. The autoradiograms in the figure depict a mammary adenocarcinoma cut in two different depths.

All analyses were performed using the MultiGauge software (Fujifilm, Japan). Since lung tumors are embedded in normal lung tissue and cannot be delineated reliably based exclusively on autoradiograms, tumors were identified and encircled by H&E images. The spatial correlation between FAZA and ^14^C-2DG was analyzed by positioning a square grid with a cell size of 0.5 x 0.5 mm on the two corresponding autoradiograms, to produce scatter plots and calculate Pearson correlation coefficients (*R*). To ensure accurate positioning of the grids, several guidance regions of interest (ROIs) were drawn encircling tissue sections and obvious landmarks, such as minor tissue fragments, holes, and folds (see Figure 1 for an example). Grid values were corrected for background signal identified in an ROI positioned outside tissue sections and normalized to background-corrected reference tissue values. Only grid-cell squares fully contained within the tumor tissue border were included, and the analysis was restricted to lesions where ≥ 10 grid-cells could fit within the tumor boundaries. Large areas of necrosis, tissue holes, and folds were omitted since they may affect assessment of the true biological relationship between tracers. The dynamic signal-range (DYR) of the tracers was assessed as the ratio between the grid-cell with the highest and lowest signal or, to increase robustness, the ratio between the mean of the 10 highest and 10 lowest (5 for the lung tumors due to their small size) grid-cells. Furthermore, the ratio of the ^14^C-2DG signal in the 10 (5 for lung) most/least hypoxic (FAZA signal strength) grid-cells was calculated (‘*in vivo* Pasteur effect’).

### Statistics/data presentation

Our data were mostly reported as scatterplots, which are ideal for visualizing the correlation between two quantitative variables. The strength of the correlation (e.g. extent of tracer overlap) was reported as the Pearson correlation coefficient (*R*). *R* and *p*-values were calculated using Sigmaplot 14.0. A *p*-value < 0.05 was considered significant.

## Results

Most mice developed tumors of a size suitable for experiments after 2–6 months. The lung and the mammary adenocarcinoma model sometimes presented with several lesions per mouse. Regardless, for the mammary model, only one tumor per mouse was included in the data analysis. For the lung model, two out of three tumors analyzed were obtained from a single mouse. The spatial correlation/overlap between hypoxic foci (FAZA) and retention of ^14^C-2DG was evaluated as described earlier (see Figure 1). A mid-sagittal brain section was used to normalize the ^14^C-2DG signal. Since the brain and muscle displayed identical signal intensity of FAZA (not shown), and brain sections are less susceptible to cutting artifacts, the brain was also used for FAZA signal normalization. The normalization process does not influence *R* but allows, when relevant, a quantitative comparison of absolute uptake of the glucose and hypoxia tracers across tumors.

**Figure 2:**
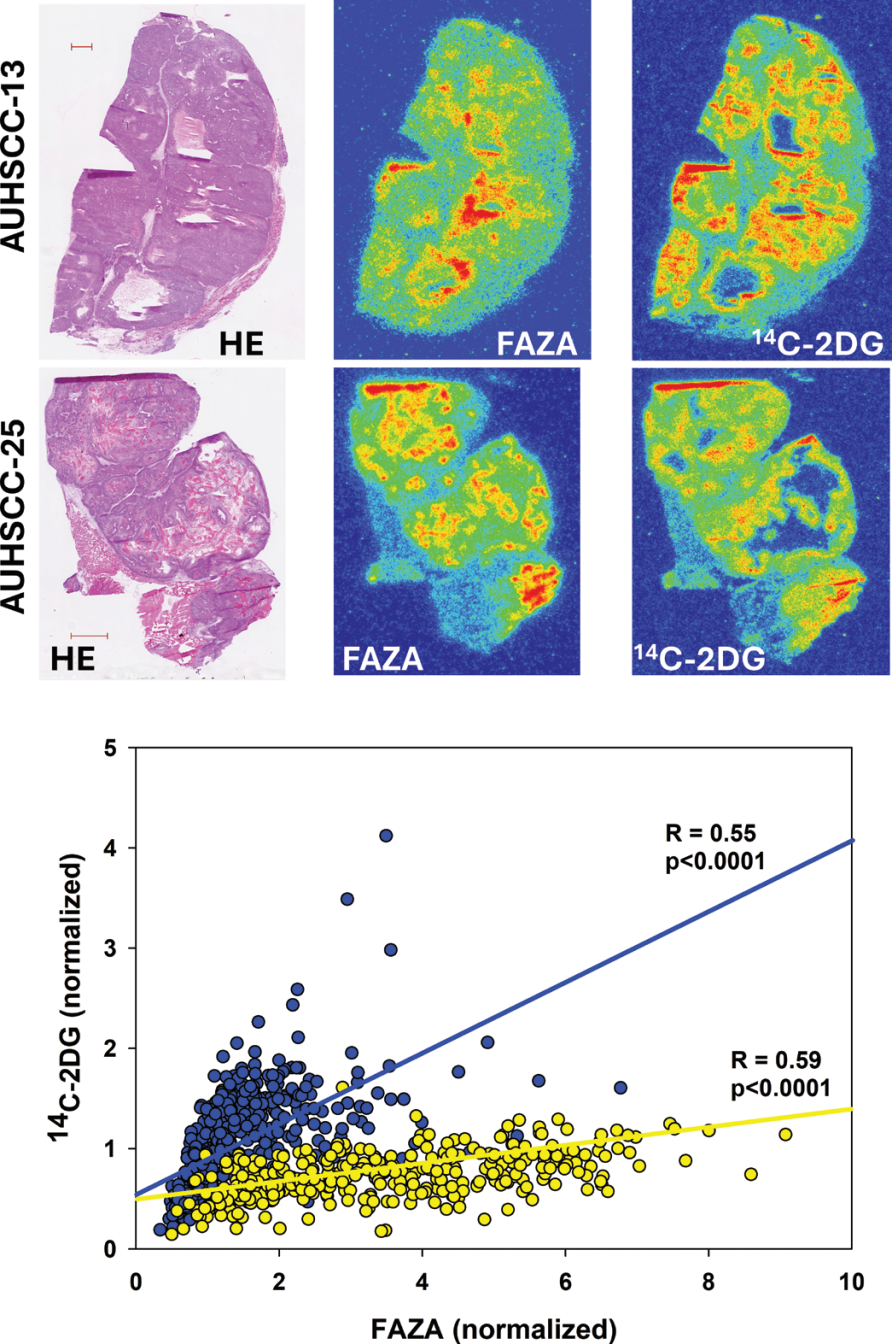
Intratumoral spatial correlation analysis between FAZA and ^14^C-2DG for two different PDX models derived from head and neck squamous cell carcinoma patients. Blue symbols/line is for the AUHSCC-13 tumor, whereas the yellow symbols/line is for the AUHSCC-25 model.

**Figure 3:**
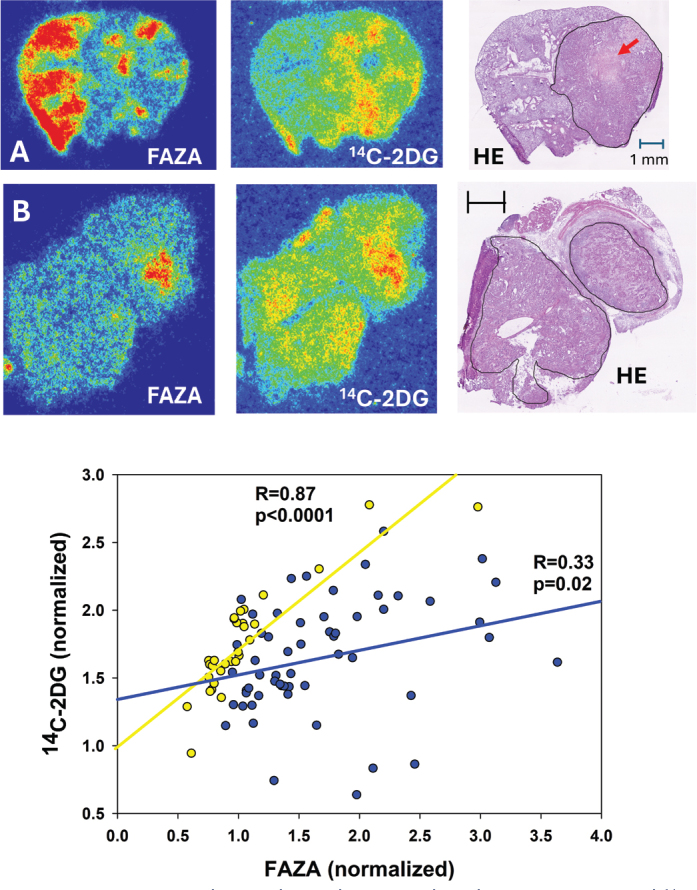
Intratumoral spatial correlation analysis between FAZA and ^14^C-2DG in two orthotopic lung adenocarcinomas. Blue points/regression line is the analysis based on tumor A, whereas yellow points/regression line is the analysis based on tumor B. Of note, there was substantial uptake of the hypoxia marker in neighbor non-transformed lung tissue in the upper tumor, which may relate to impaired blood flow caused by the tumor. The red arrow shows an area of necrosis.

**Figure 4:**
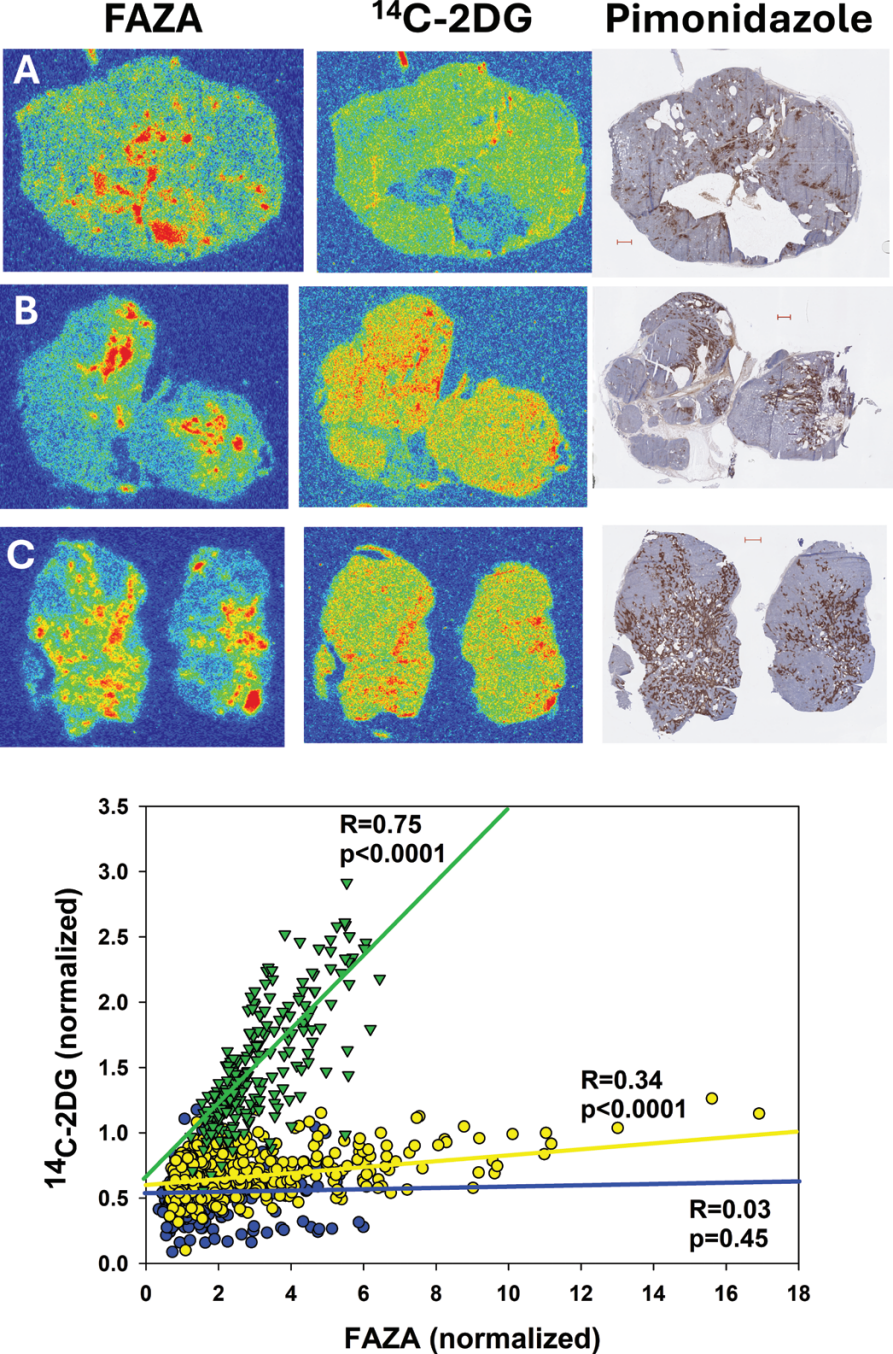
Intratumoral spatial correlation analysis between FAZA and ^14^C-2DG in three orthotopic mammary adenocarcinomas reveals substantial tumor-to-tumor variability. Blue, yellow, and green points/lines represent tumors A, B, and C, respectively. Another example of a mammary adenocarcinoma with a strong spatial correlation between the two tracers is shown in Figure 1. Subsequent immunostainings showed that FAZA, as expected, distributes as pimonidazole.

Data for individual tumors are summarized in Table 1. In AUHSCC-13 tumors (*n* = 4), *R* ranged from 0.55 to 0.82 (average: 0.64), which was rather similar to AUHSCC-25 tumors (*n* = 4) where *R* varied from 0.48 to 0.65 (average: 0.59). An example for each model is shown in Figure 2. In the lung tumor model (Figure 3), tumors were typically small, and only three tumors met the inclusion criteria (the presence of FAZA-retaining foci and a section size that accommodates at least 10 grid-cells). In two tumors, there was a strong spatial overlap between the two tracers (*R* > 0.8), whereas the third tumor exhibited a weaker (*R* = 0.33), albeit still significant, correlation. Mammary tumors (*n* = 7) displayed large tumor-to-tumor variability with *R* varying between 0.03 and 0.82 (average: 0.48), although *R* was above 0.6 in four tumors. Selected examples are shown in figure 4.

**Table 1:** Summary of all data for four different tumor models.

Analysis	FAZA	^14^C-2DG	Ratio (1 pixel)	FAZA	^14^C-2DG	Ratio (10 pixels)	*In vivo* Pasteur effect	Pearson
Parameter	DYR (pixel_1_)	DYR (pixel_1_)	FAZA_DYR_/^14^C-2DG_DYR_	DYR (pixel_10_)	DYR (pixel_10_)	FAZA_DYR_/^14^C-2DG_DYR_	^14^C-2DG_FAZA, max_/^14^C-2DG_FAZA, min_	R
Tumor								
HER2+	17.6	4.4	4.0	11.6	2.4	4.8	1.46	0.63
HER2+	26.2	4.0	6.6	16.2	3.0	5.3	1.62	0.34
HER2+	17.0	13.5	1.3	10.1	5.7	1.8	1.14	0.03
HER2+	9.9	2.2	4.5	4.4	1.8	2.5	1.32	0.11
HER2+	5.2	5.2	1.0	3.8	3.2	1.2	2.17	0.75
HER2+	15.5	3.3	4.7	11.4	2.4	4.8	2.39	0.67
HER2+	55.4	5.3	10.5	28.6	3.4	8.5	2.49	0.82
**Mean**	**21.0**	**5.4**	**4.5**	**13.5**	**3.1**	**4.1**	**1.80**	**0.48**
Lung/CRISPR	5.4	3.2	1.7	2.7*	2.0*	1.4	1.77	0.87
Lung/CRISPR	9.6	3.5	2.8	6.2*	2.7*	2.3	2.28	0.81
Lung/CRISPR	5.0	3.9	1.3	3.6*	2.5*	1.4	1.36	0.33
**Mean**	**6.7**	**3.5**	**1.9**	**4.2**	**2.4**	**1.7**	**1.80**	**0.67**
AUHSCC-13	23.6	13.4	1.8	18.5	6.7	2.7	4.60	0.82
AUHSCC-13	20.1	21.8	0.9	9.5	9.0	1.1	3.54	0.55
AUHSCC-13	14.9	11.1	1.3	6.9	5.2	1.3	2.53	0.62
AUHSCC-13	17.1	18.8	0.9	10.7	8.0	1.3	3.26	0.56
**Mean**	**18.9**	**16.3**	**1.2**	**11.4**	**7.2**	**1.6**	**3.48**	**0.64**
AUHSCC-25	26.5	11.7	2.3	12.3	6.2	2.0	3.2	0.59
AUHSCC-25	14.7	7.4	2.0	9.7	4.7	2.1	2.3	0.64
AUHSCC-25	18.6	8.9	2.1	10.3	5.8	1.8	2.5	0.48
AUHSCC-25	79.4	19.4	4.1	23.5	9.5	2.5	4.8	0.65
**Mean**	**34.8**	**11.8**	**2.6**	**13.9**	**6.6**	**2.1**	**3.22**	**0.59**

The dynamic range (DYR) was calculated as the ratio between the most and least intense grid-cell or as the average of the 10 most or least intense grid-cells (to increase robustness). Due to their smaller size, we instead used the 5 most/least intense grid-cells for lung tumors (*). The ‘*in vivo* Pasteur effect’ was calculated as the average ^14^C-2DG signal in the 10 most hypoxic grid-cells (as based on FAZA) divided by the average ^14^C-2DG signal in the 10 least hypoxic grid-cells. Pearson correlation coefficients (*R*) were calculated based on scatterplot analyses as exemplified in various figures.

For most tumors, the dynamic range (DYR) for FAZA was greater than that for ^14^C-2DG (Table 1), suggesting that although hypoxia stimulates FDG retention, relative changes may be more modest than those observed when using true hypoxia-selective tracers. The *in vivo* Pasteur effect (fold-change in ^14^C-2DG between non-hypoxic and the most hypoxic areas) was highest in the PDX models.

## Discussion and conclusion

Overall, results were mixed. In two subcutaneous HNSCC PDX models and an orthotopic lung tumor model, the *R* value typically ranged between 0.5 and 0.8. Tumor-to-tumor variability was much greater in mammary adenocarcinomas, ranging from 0.03 to 0.82. Our results suggest that FDG-PET, in some tumors, may be suitable for mapping hypoxia. Intertumor differences in the mutational landscape may be responsible for the variability, and future work unraveling the link between mutations and the strength of interrelationship between FDG and hypoxia-PET is pivotal, since it could allow gene-based identification of tumors where FDG-PET can substitute hypoxia-PET. Alternatively, in a treatment scenario where multiple PET scans are required (e.g. to assess the dynamics of hypoxia), such tumors may be identified from a pretreatment FAZA/FDG dual-tracer PET scan, followed by FDG-PET when deemed a suitable alternative.

We have developed and refined methodologies that allow PET-mimicking dual-tracer autoradiographic analyses of FAZA and ^14^C-2DG in small (e.g. lung) tumors, with a size that makes PET studies futile even when using high-resolution animal scanners. ^14^C-2DG is the most widely used glucose analogue in preclinical research since it can be stored for extended periods and does not require an on-site cyclotron. The trapping mechanism for FDG and ^14^C-2DG is identical, but the two tracers differ chemically. Regardless, it has previously been shown by others [21] and us (unpublished) that the two tracers distribute similarly intratumorally (*R*≈0.95), making ^14^C-2DG a fully suitable mimic for FDG. Importantly, our methodology (1) enables intratumoral spatial comparisons of different tracers with negligible co-registration inaccuracies, (2) fully circumvents potential time-to-time differences in multi-tracer PET studies caused by changes in the growing and dynamic tumor tissue, which could lead to biased and faulty conclusions where true associations are underestimated, and (3) allows a direct comparison of PET-relevant tracers with the underlying tumor characteristics visualized immunohistochemically. The method can easily be extended to other models and tracer combinations.

Intravenous (IV) tracer administration via the tail vein and normalization to injected dose/body mass is considered the state of the art method. However, the accuracy of this approach may be compromised by variable (unknown!) levels of competing glucose in plasma, differences in physical activity, or imperfect (often unnoticed) tail vein injections. Indeed, we have previously demonstrated that intraperitoneal tracer administration and normalization of tumor FDG retention to whole-brain tracer retention (FDG-PET/MRI) are superior to traditional PET-based quantification of standardized uptake value (SUV) metrics in IV tracer-injected animals since differences in tracer bioavailability/tracer input function and plasma glucose will influence tracer retention in the two tissues to a similar extent [22]. In addition, the larger volume that can be administered IP allows injection of PET tracers and pimonidazole in a single bolus. To apply this PET-image quantification approach in an autoradiography-based setting, we normalized all autoradiographic data by including a mid-sagittal brain section, since this is the only symmetry-axis that can be identified easily and reproducibly. Obviously, our normalization method does not affect *R*, but it allows comparisons of absolute tracer retention across tumors when relevant.

Mitochondrial adenosine triphosphate (ATP) production is compromised under hypoxia, which may be compensated for by increased glycolytic ATP synthesis (anaerobic glycolysis). Considering the all-dominant role FDG-PET plays diagnostically, the possible use of FDG-PET as a surrogate for the assessment of hypoxia has received considerable interest. However, the use of glucose is influenced by a plethora of tumor characteristics other than oxygenation, including cellularity, immune-cell infiltration, proliferative rate, overall metabolic rate, blood flow, tumor microenvironment (e.g. pH), and necrosis. Accordingly, it is unsurprising that traditional FDG-PET metrics like SUVmax, SUVpeak, or SUVmean generally correlate poorly with overall retention of more hypoxia-selective tracers such as FMISO [23] and FAZA [24], or expression levels of endogenous hypoxia markers including HIF1α or downstream targets [25]. In recent years, a growing interest for more detailed textural analysis of FDG-PET to define and quantify intratumoral voxel heterogeneity has received considerable interest. Understanding the relationship between tracer retention and underlying pathophysiology on a voxel-by-voxel basis may improve tumor characterization, and thus treatment-decision-making. Indeed, hypoxia may be an important, or even the major, driver of the observed intratumoral heterogeneity. If so, FDG-PET maps may provide useful information on hypoxia, based on oxygenation-driven glucose consumption, which could guide dose-painting in radiotherapy, even though global or maximal FDG uptake (i.e. SUV metrics) is poorly correlated to tumor oxygenation [26]. Of note, the inherent glycolytic phenotype in tumor cells (aerobic glycolysis/Warburg effect) may overall dampen hypoxia-driven changes (the Pasteur effect), but whether this *per se* makes the general use of FDG-PET in hypoxia-imaging futile is not clear. Accordingly, numerous clinical studies have compared the distribution of hypoxia-selective tracers (e.g. FMISO, FAZA, and HX4) with FDG-maps. Results are mixed and may be disease-specific and should thus be addressed and discussed separately.

Thorwarth et al. showed that the spatial distribution-similarity of FMISO and FDG varied widely in 12 HNSCC patients [11]. Interestingly, the authors concluded that a poor and/or noisy regression plot may predict poor outcome, possible due to an aggressive unbalanced growth in those tumors, suggesting a role for dual-tracer examinations for treatment decision-making. In another study including 26 HNSCC patients, an average Pearson correlation coefficient of 0.62 between FDG and FMISO concentrations was observed [13]. In a third study based on 20 patients, it was concluded that there was a moderate, yet variable, correlation between FMISO and FDG in the primary tumors but not in metastatic lymph nodes [12]. To what extent this patient-to-patient and study-to-study discordance relates to true biological differences or rather reflects technical limitations in clinical settings, where scans are time-separated (by one or more days) and co-registration issues may be prevalent, remains unclear. This putative inherent methodological weakness is underscored by two FMISO-PET repeatability studies in comparable HNSCC patient groups, where one study concluded that in half of the patients, there was considerable scan-to-scan discordance in the intratumoral distribution of FMISO [27], whereas the other study reported an excellent agreement between two time-separated scans in terms of traditional measures (tumor-to-muscle ratio) and the intratumoral tracer distribution pattern [28]. Such differences raise a red flag since technical issues including co-registration accuracy may lead to erroneous and biased conclusions. Taken together, our preclinical data fit reasonably well with clinical studies and highlight that some of the variability in clinical trials may relate to technology and biological differences between time-separated scans.

Based on the analysis of three orthotopic lung tumors, there was a reasonable, yet variable, spatial correlation between FAZA and ^14^C-2DG, which agrees with clinical trials. Indeed, it was shown that FMISO adds little novel information when it comes to the target definition in lung cancer patients, and FDG may thus be a valuable substitute to guide dose-painting strategies designed to overcome hypoxia-related resistance [9]. In another study, a reasonable spatial correlation was observed in most tumors when comparing FDG to the hypoxia tracer HX4 [29]. In contrast, Bollineni et al. [8] reported a poor spatial correlation between FDG and FAZA-PET, but only regions/voxels above a certain FDG-PET-defined threshold (target volume) were included, which may cause a bias and weakened correlations. In the present study, we inactivated the tumor suppressor LKB1, which is mutated in 35% of lung adenocarcinomas [30]. AMP-activated kinase is a target of LKB1 and a master regulator of energy metabolism under energetic stress including low glucose and hypoxia [31]. Loss of LKB1 has been linked to a more glycolytic phenotype (Warburg effect) and increased vulnerability toward energetic stress like hypoxia, which could lead to an uncoupling of local glucose use and oxygenation, which contrasts our *in vivo* findings. Unfortunately, there is little *in vivo* evidence that decisively couples LKB1 status to oxygenation-dependent stimulation of glycolysis or the extent and consequences (e.g. prognosis and treatment resistance) of hypoxia. Indeed, we previously showed that the LKB1-mutated cervical squamous cell carcinoma cell line SiHa has a distinct non-glycolytic phenotype *in vitro* [32, 33] as well as *in vivo* [34]. Interestingly, a clinical study showed that the incidence of treatment failure was higher in patients with low tumor LKB1 expression, and tumor recurrence was always observed within the radiation field, suggesting elevated radioresistance, but whether this may relate to the presence of viable hypoxic cells was not investigated [35]. Clearly, much more research on this intriguing subject is justified, and the orthotopic model is valuable.

Although hypoxia-PET and other methodologies have demonstrated the presence of hypoxia in mammary adenocarcinoma patients, there is, to the best of our knowledge, only one study that compared the distribution patterns of hypoxia-PET and FDG-PET [36]. The authors reported a Pearson correlation of 0.51 but did not provide data on patient-to-patient variability.

Besides a strong spatial relationship (high R), reliable and robust PET-generated hypoxia maps also depend on intratumor tracer signal heterogeneity (i.e. DYR), which was considerable for both tracers (Table 1). FAZA typically displayed higher DYR values, but this may relate to the use of rodents, where wash-out of unbound tracer is more effective, leading to higher image contrast [37]. Of note, the glucose-tracer retention ratio between non-hypoxic and most hypoxic areas (the ‘*in vivo* Pasteur effect’, Table 1) was highest and similar in the 2 HNSCC models, but it is reasonable in lung cancers, considering that FAZA hotspots were less intense in this model. Differences in the *in vivo* Pasteur effect may reflect underlying metabolic differences, such as the inherent relative reliance on aerobic glycolysis [32, 34], and it would be of interest to unravel links between the underlying mutational landscape and the strength of the linkage between regional glucose use and oxygenation. The highly versatile CRISPR/Cas9 model (allows targeted mutations) and the Her2+ model, where intertumor mutational heterogeneity is expected, are ideal models for such studies.

## Conclusion

Clinical trials comparing the relationship between the retention of FDG and hypoxia-PET remain inconclusive. Furthermore, preclinical rodent dual-tracer PET scans are often not feasible due to their small size and moving artifacts (lungs). Therefore, we developed a powerful autoradiography-based experimental tool to assess tracer uptake patterns in clinically relevant PDX and orthotopic mammary and lung tumors that would be unachievable to conduct based on PET imaging. Our methodology is ideal to further address similarities and differences between FDG-PET and hypoxia-PET in dose-escalation settings.

## Data Availability

Data are available upon request (morten@oncology.dk).
